# Development, Characterization, and Radiation Dosimetry Studies of ^18^F-BMS-986229, a ^18^F-Labeled PD-L1 Macrocyclic Peptide PET Tracer

**DOI:** 10.1007/s11307-023-01889-4

**Published:** 2023-12-20

**Authors:** Joonyoung Kim, David J. Donnelly, Tritin Tran, Adrienne Pena, Andrea Olga Shorts, Thomas V. Petrone, Yunhui Zhang, Kenneth M. Boy, Paul M. Scola, Daniel J. Tenney, Michael A. Poss, Matthew G. Soars, Samuel J. Bonacorsi, Erin L. Cole, Diederik J. Grootendorst, Patrick L. Chow, Nicholas A. Meanwell, Shuyan Du

**Affiliations:** Bristol Myers Squibb Research and Early Development, P.O. Box 4000, Princeton, NJ 08543 USA

**Keywords:** PD-1:PD-L1 checkpoint inhibitor, ^18^F-labeled macrocyclic peptide, Preclinical PET imaging

## Abstract

**Purpose:**

In cancer immunotherapy, the blockade of the interaction between programmed death-1 and its ligand (PD-1:PD-L1) has proven to be one of the most promising strategies. However, as mechanisms of resistance to PD-1/PD-L1 inhibition include variability in tumor cell PD-L1 expression in addition to standard tumor biopsy PD-L1 immunohistochemistry (IHC), a comprehensive and quantitative approach for measuring PD-L1 expression is required. Herein, we report the development and characterization of an ^18^F-PD-L1-binding macrocyclic peptide as a PET tracer for the comprehensive evaluation of tumor PD-L1 expression in cancer patients.

**Procedures:**

^18^F-BMS-986229 was characterized for PD-L1 expression assessment by autoradiography or PET imaging. ^18^F-BMS-986229 was utilized to evaluate tumor PD-L1 target engagement in competition with a macrocyclic peptide inhibitor of PD-L1 (BMS-986189) over a range of doses using PET imaging. A whole-body radiation dosimetry study of ^18^F-BMS-986229 in healthy non-human primates (NHPs) was performed.

**Results:**

*In vitro* autoradiography showed an 8:1 binding ratio in L2987(PD-L1 +) vs. HT-29 (PD-L1-) tumors, more than 90% of which could be blocked with 1 nM of BMS-986189. *Ex vivo* autoradiography showed that ^18^F-BMS-986229 detection was penetrant over a series of sections spanning the entire L2987 tumor. *In vivo* PET imaging in mice demonstrated a 5:1 tracer uptake ratio (at 90–100 min after tracer administration) in L2987 vs. HT-29 tumors and demonstrated 83%-93% specific binding of BMS-986189 within those dose ranges. In a healthy NHP dosimetry study, the resultant whole-body effective dose was 0.025 mSv/MBq.

**Conclusion:**

^18^F-BMS-986229 has been preclinically characterized and exhibits high target specificity, low background uptake, and a short blood half-life supportive of same day imaging in the clinic. As the PET tracer, ^18^F-BMS-986229 shows promise in the quantification of PD-L1 expression, and its use in monitoring longitudinal changes in patients may provide insights into PD-1:PD-L1 immuno-therapy treatment outcomes.

The rapidly evolving fields of tumor immunology and cancer immunotherapy have led to the U.S. Food and Drug Administration’s approval of immunotherapies directed to T cell checkpoints [1–3]. Inhibition of the immune response checkpoint mediated by programmed death-1 receptor (PD-1) and its ligand (PD-L1) plays a critical role in improving the prognosis of patients with multiple tumor types [4]. Inhibition of the interaction between PD-1 and PD-L1 can enhance T-cell responses and mediate antitumor activity [5]. Human monoclonal antibodies directed toward either PD-1 (nivolumab) [6, 7] or PD-L1 [8] have shown clinical efficacy against multiple tumor types. In some tumor types, it has been reported that PD-L1 expression by the patient’s tumor increases the probability that a patient will respond favorably to PD-1:PD-L1 blockade [9–11]. It is demonstrated that PD-L1 expression can be a factor which closely correlates with response to anti-PD-1:PD-L1 checkpoint inhibitor.

In the early clinical development of an anti-PD-1 antibody, the PD-L1 IHC analysis of tumor biopsies suggested a relationship between PD-L1 expression on tumor cells and an objective response to PD-1 pathway therapy. Nine out of 25 patients with human non-small cell lung cancer (NSCLC), melanoma, or renal cell cancer with ≥ 5% of PD-L1 positive tumor cells had an objective response rate of 36%, while the remainder (PD-L1 < 5%) had no objective response [10]. However, while tumor biopsy IHC is still the standard method to measure PD-L1 expression, it has clear limitations with respect to its invasive nature and intra-tumoral heterogeneity (only a small part of a single tumor is typically sampled). Additionally, as pretreatment baseline biopsies are most used to assess tumor PD-L1 expression, the effect of checkpoint therapy on PD-L1 expression during treatment is not usually monitored. These limitations warrant a need for non-invasive methods of the comprehensive and quantitative assessment of PD-L1 expression and its dynamic change in tumors and metastasis, which may provide better insights for predicting tumor response and monitoring the treatment efficacy of immunotherapies.

It has been demonstrated that radiolabeled PD-1 or PD-L1 antibodies can be used to non-invasively assess PD-1 or PD-L1 expression in NHPs, human tumor xenografts, and syngeneic tumor models [12–17]. Although radiolabeled antibodies are used for imaging tumor-specific proteins, a longer clearance time of radiolabeled antibodies is required for enhanced image contrast and lesion detection. In this regard, PD-L1 tracers with low molecular weight, faster clearance, short-lived radioactivity, that are relatively easy to radio-synthesize with high specific activity are desirable for clinical application due to the “same day” imaging feasibility and wider clinical availability. ^18^F-BMS986192, an anti-PD-L1 adnectin derived from the 10th type III domain of human fibronectin (~ 10 kDa) was reported as the first PD-L1 PET tracer for same-day imaging and has been successfully translated into clinical study application [17, 18]. However, its synthesis remains challenging and is isolated in modest yields.

This study reports the characterization of ^18^F-BMS-986229, an anti-PD-L1 macrocyclic peptide (2 kDa), as a novel PD-L1 PET tracer with the potential for a binding profile, tumor penetration, and clearance *in vivo*. And for clinical use of PET tracer, whole-body radiation dosimetry via PET imaging is assessed in healthy NHPs.

## Materials and Methods

All animal studies were conducted according to the protocols approved by Bristol Myers Squibb’s Animal Care and Use Committee. In addition, BMS-986189, which is prepared from the same chemotype series as the PET tracer, was used to block the binding of the PET tracer to PD-L1 and its target engagement. It had been shown to bind to human PD-L1 (K_D_ < 10 pM via surface plasmon resonance, SPR) with NHP PD-L1.

### Synthesis of ^18^F-BMS-986229

The synthesis of ^18^F-BMS-986229 has been described in recent published paper [19]. Summarized here, ^18^F-BMS-986229 was prepared via a copper-mediated azide-alkyne cycloaddition reaction between ^18^F-BMT-187144, a novel azide containing prosthetic group discovered at Bristol Myers Squibb, and an advanced alkyne containing macrocyclic peptide, a process that proceeded in high radiochemical yield (17% non-decay corrected yield), high chemical and radiochemical purity (> 90%) with high molar activity (229.4 MBq/nmol).

### Cell Lines and Tumor Xenografts

L2987 is a PD-L1 positive human lung adenocarcinoma cell line, while HT-29 is a PD-L1 negative human colorectal adenocarcinoma cell line (American Type Culture Collection) [17]. L2987 cells were cultured in Roswell Park Memorial Institute medium supplemented with 10% fetal bovine serum (FBS), while the HT-29 cells were cultured in Minimum Essentia Media supplemented with 10% FBS. All cells were maintained in a humidified incubator at 37 °C with 5% CO_2_.

Anti-human CD274-APC (eBioSciences cat#17–5983-42) was used to quantify PD-L1 density on the tumor cell lines. PD-L1 copy number calculations were dependent on standard curves of Quantum™ Simply Cellular® beads (Bangs Labs, Fishers, IN) using a procedure outlined by the manufacturer. All FACS analyses were performed using Forecyt software (Intellicyt Corp, Albuquerque, NM). The copy numbers of PD-L1 expressed by the HT-29 and L2987 cells were 1,629 and 106,000 copies per cell (very near the limit of detection of 859 copies), respectively. 

### *In Vitro *and *Ex Vivo* Characterizations of ^18^F-BMS-986229

#### *In Vitro* Autoradiography

HT-29 and L2987 xenograft tumors were harvested approximately 2–3 weeks from the implantation date. Tissue sections of 10 µm thickness were prepared and thaw–mounted onto glass slides. Six different fresh-frozen human NSCLC tumor samples were also obtained (Asterand Biosciences) [17]. HT-29 and L2987 tissue slides in a glass chamber were co-incubated with 40 mL of Tween buffer solution plus BMS-986189 at concentrations of 0, 1, or 10 nM, and 0.25 nM of ^18^F-BMS-986229. Human NSCLC tumor tissues were co-incubated with 40 mL of Tween buffer solution (50 mM HEPES, 10 nM NaCl, pH = 7.4 and 0.05% Tween-20) containing 0, 0.05, 0.3, 1, or 10 nM of BMS-986189, and 0.25 nM of ^18^F-BMS-986229. These slides were incubated for 1 h at room temperature and then were rinsed 4 times with an ice-cold Tween wash buffer. Air-dried slides were exposed by placing the slides onto a storage phosphor screen (BAS-SR 3545S, GE healthcare Biosciences) overnight at room temperature. The imaging plate was scanned the following morning using a phosphor-imager (Fujifilm Fluorescent Image Analyzer, FLA-9000) and image analysis was performed using Multi-Gauge software (FujiFilm, Ltd.). Regions of interest (ROIs) were drawn to surround the entire tumor section in all study groups, and total radioligand binding values were determined. Intensity is measured by photo-stimulated luminescence per area (PSL/mm^2^).

#### *Ex Vivo* Autoradiography

HT-29 and L2987 tumors were collected from two different mice after PET imaging was complete (~ 110 min after tracer injection) to examine tracer infiltration into the tumor. Tissue sections of 10 µm thickness were prepared at 200 μm intervals. Air-dried slides were exposed by placing the slides onto an imaging plate overnight at room temperature. The rest of the process was the same as described above.

### Preclinical PET Imaging in Tumor-Bearing Mice

#### Tumor-Bearing Mice Model

*In vivo* studies were performed by implanting tumor xenografts in 5–6 weeks old female athymic nude mice (Charles River Laboratories). Bilateral tumor xenografts were established by subcutaneous inoculation of HT-29 (1.5 × 10^6^ cells) and L2987 (4 × 10^6^ cells) in contralateral shoulders. All cells were prepared at the appropriate concentration in Hank’s balanced salt solution in a total injection volume of 0.2 mL. When tumor-caliper measurement reached approximately 200–400 mm^3^ (~ 2–3 weeks after cell implantation), animals were selected for imaging. Body weight and tumor size were measured on the day of imaging and recorded prior to imaging.

#### PET/CT Imaging

Mice were placed into a custom plexiglass 4-chamber mouse hotel (BMS Applied Biotechnology) under 1–1.5% isoflurane inhalant anesthesia, delivered via nosecone at 2 L/min in 100% O_2_. The custom mouse hotel allowed co-registration of anatomical structures and tumors in both the microCT (XSPECT, TriFoil Imaging, Inc.) and microPET (MicroPET® FOCUS 120™, Siemens Preclinical Solutions, Knoxville, TN). The scanning region extended from the eyes to the tail of the mouse (Axial field of view = 7.6 cm). For attenuation correction, a 10-min transmission scan was performed using a ^57^Co source followed by an emission scan. ^18^F-BMS-986229 was administered via the tail vein 30 min after subcutaneous administration of BMS-986189 or vehicle. PET images were reconstructed using a filtered back-projection (FBP) algorithm corrected for attenuation. ROIs encompassing tumors and background tissue (e.g., muscle) were manually drawn in axial co-registered PET/CT images. Image analysis was performed using the ASIPro software (Siemens Healthineers) and a standardized uptake value (SUV) based on body weight was calculated.

#### Biodistribution Study with ^18^F-BMS-986229

For investigating the distribution of tracer and specific binding upon administration of BMS-986189 at a dose of 60 mg/kg, 120 min-dynamic PET imaging in tumor-bearing mice was acquired using 10 frames × 60 s and 11 frames × 600 s. In the vehicle group, four tumor-bearing mice were used (body weight: 22.8 ± 2.4 g, injection dose: 4.4 ± 0.4 MBq, tracer mass: 0.98 ± 0.48 µg/kg, tumor volume: 233.1 ± 59.1 mm^3^ in HT-29 and 453.9 ± 69.9 mm^3^ in L2987). In the BMS-986189 blocking group, four tumor-bearing mice were used (21.7 ± 1.6 g, 4.5 ± 0.2 MBq, 1.04 ± 0.46 µg/kg, 218.5 ± 72.4 mm^3^ in HT-29 and 391.9 ± 176.2 mm^3^ in L2987). Data are shown as mean ± standard deviation.

#### Blocking Study with BMS-986189

For measuring target engagement, multiple blocking doses were used (0.05, 0.1, 0.3, 2, 7, 15, 30, 60 mg/kg of BMS-986189) as well as saline, injected in a volume by 1 mL/kg. After injecting the tracer, mice were allowed to move freely in the cage for approximately 75 min. A 10 min-static PET image was acquired 90 min after tracer IV injection in tumors-bearing mice: *N* = 58, 23.0 ± 2.2 g, 3.0 ± 0.6 MBq, 4.45 ± 2.07 µg/kg, 124.3 ± 63.7 mm^3^ in HT-29 and 271.4 ± 152.0 mm^3^ in L2987.

### Radiation Dosimetry in Healthy Non-Human Primates

#### PET Imaging

Whole-body dynamic PET imaging of cynomolgus macaques (2 females and 2 males, 3.7 ± 0.4 kg) was obtained using a microPET® F220™ camera (Siemens Preclinical Solutions, Knoxville, TN). The NHPs were placed in a Plexiglas custom imaging bed (BMS Applied Biotechnology), while under ~ 1–1.5% isoflurane inhalant anesthesia delivered in 100% O_2_ at ~ 1 L/min via positive pressure ventilation. Whole-body PET images were acquired with a total of 8 overlapping (1.5 cm) scan positions moving progressively down from the top of the NHP’s head (position 1) to its hind limbs (position 8) due to the limitation of the axial field of view (7.6 cm). Imaging began with a whole-body transmission scan using a ^57^Co source (10 min/bed position) for attenuation correction. Emission imaging began immediately after administration of the radiotracer (30.6 ± 4.5 MBq) via the saphenous vein catheter and continued for approximately 4.5 h. Emission data for all NHPs were collected for 1 min per bed position for the first full body scan, 2 min/bed position for the second full body scan, 3 min/bed position for the third full body scan, and 5 min/bed position for the remaining passes, resulting in 8 whole-body images. All images were reconstructed with the FBP algorithm and attenuation correction. Whole-body PET images were formed by stitching the individual bed positions together using in-house developed stitching software and AMIDE software version 0.9.1 [20].

#### Analysis of Images

ROIs encompassing source organs that were visually identifiable on PET images (heart, liver, spleen, gallbladder, kidney, and urinary bladder) were manually drawn, and the non-decay corrected activity concentration at each time point was extracted. Data were then extrapolated up to 12 h by assuming that any further decline in activity occurred by physical decay without any biological redistribution or clearance. The area under the curve (AUC) of each organ was calculated by the trapezoidal method. The calculated AUCs for each organ were then converted to human values by multiplication of average human organ volumes [21] followed by multiplication of (b_m_/b_h_), where b_m_ and b_h_ are the body weight of NHP and average human subject, respectively. Finally, the AUC units were normalized with injected radioactive dose. The AUC of the fractional activity corresponds to the residence times for the source organs. The residence time from all source organs was summed and subtracted from the fixed theoretical value of total residence time for ^18^F (t_1/2_/ln 2 ≈ 2.64 h) to calculate the residence time of the remainder of the body. Subsequently, the calculated residence times were entered into OLINDA/EXM 1.0 [22] using the 74 kg male phantom model for the male subjects and the 57 kg female phantom model for the female subjects [23, 24].

## Results

### *In Vitro *and *Ex Vivo* Autoradiography

**Fig. 1:**
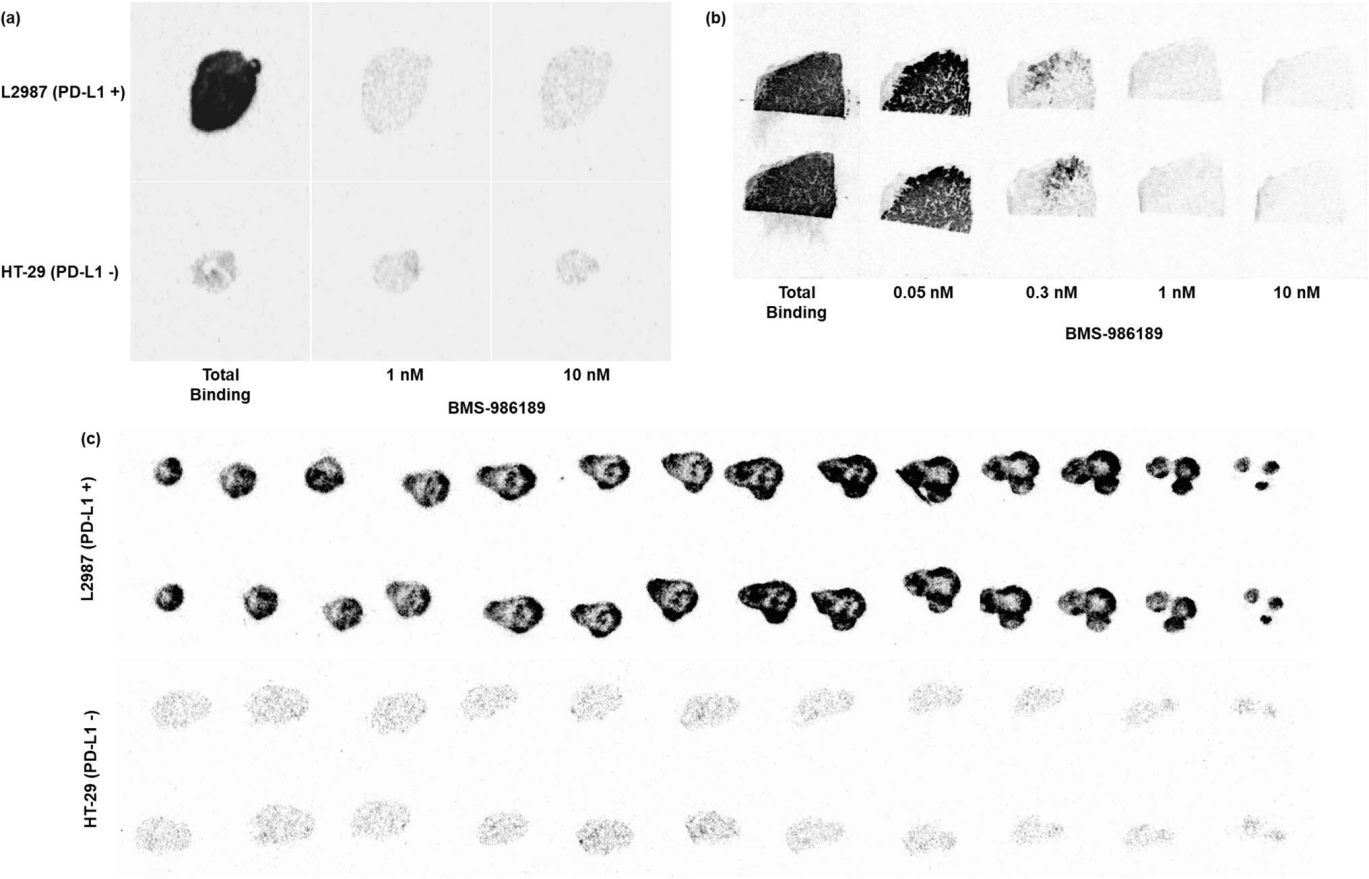
*In vitro* and *ex vivo* autoradiography in human xenograft tumors and human NSCLC tissues. (**a**) *In vitro* autoradiographic images in xenograft tumor tissues, (**b**) *In vitro* autoradiographic images in human NSCLC tissues, (**c**) *Ex vivo* autoradiographic images in xenograft tumor tissues

To examine *in vitro* binding and specificity, tumor sections from L2987 (PD-L1 +) and HT-29 (PD-L1-) xenografts were incubated with BMS-986189 (0, 1, or 10 nM) and 0.25 nM of ^18^F-BMS-986229. The intensities of the L2987 and HT-29 tumor ROIs were 665.4 ± 116.2 PSL/mm^2^ and 85.3 ± 9.8 PSL/mm^2^, respectively. The binding ratio of tracer in L2987 to HT-29 tumor sections was demonstrated to be 8:1. With co-incubation of BMS-986189 at 1 nM and 10 nM, respectively, there were 93.3% and 93.1% reductions in the binding of the tracer in the L2987 tumors, as well as 40.7% and 42.9% reductions in the HT-29 tumors, from total binding (0 nM of BMS-986189) (Fig. 1A). This indicated the binding was specific for PD-L1.

Next, the binding of the PET tracer within human NSCLC biopsies was explored, which suggested a very high level of PD-L1 expression (Fig. 1B). Tumor sections were incubated with BMS-986189 (0, 0.05, 0.3, 1, or 10 nM) and 0.25 nM of ^18^F-BMS-986229. The intensity for total binding (0 nM of BMS-986189) in the autoradiographic image was 316.8 ± 9.7 PSL/mm^2^. Upon co-incubation with BMS-986189 at different concentrations, autoradiographic image signals were 276.1 ± 47.6 PSL/mm^2^ at 0.05 nM (~ 13% blocking of total binding), 66.7 ± 7.0 PSL/mm^2^ at 0.3 nM (~ 79% blocking of total binding), 25.5 ± 1.9 PSL/mm^2^ at 1 nM (~ 92% blocking of total binding), or 16.3 ± 0.3 PSL/mm^2^ at 10 nM (95% blocking of total binding), respectively. Consequently, approximately 1 nM of BMS-986189 blocked over 90% of the tracer binding.

To ensure intra-tumoral tracer distribution, tumors were excised at approximately 110 min after tracer administration (at the end of PET imaging) and sectioned completely across their largest diameter to provide slides for *ex vivo* autoradiography. Tracer uptake throughout the tumor sections was observed for the L2987 tumor versus the HT-29 tumor (Fig. 1C), showing 10:1 uptake ratio and tumor penetration of ^18^F-BMS-986229.

### PET Imaging

**Fig. 2:**
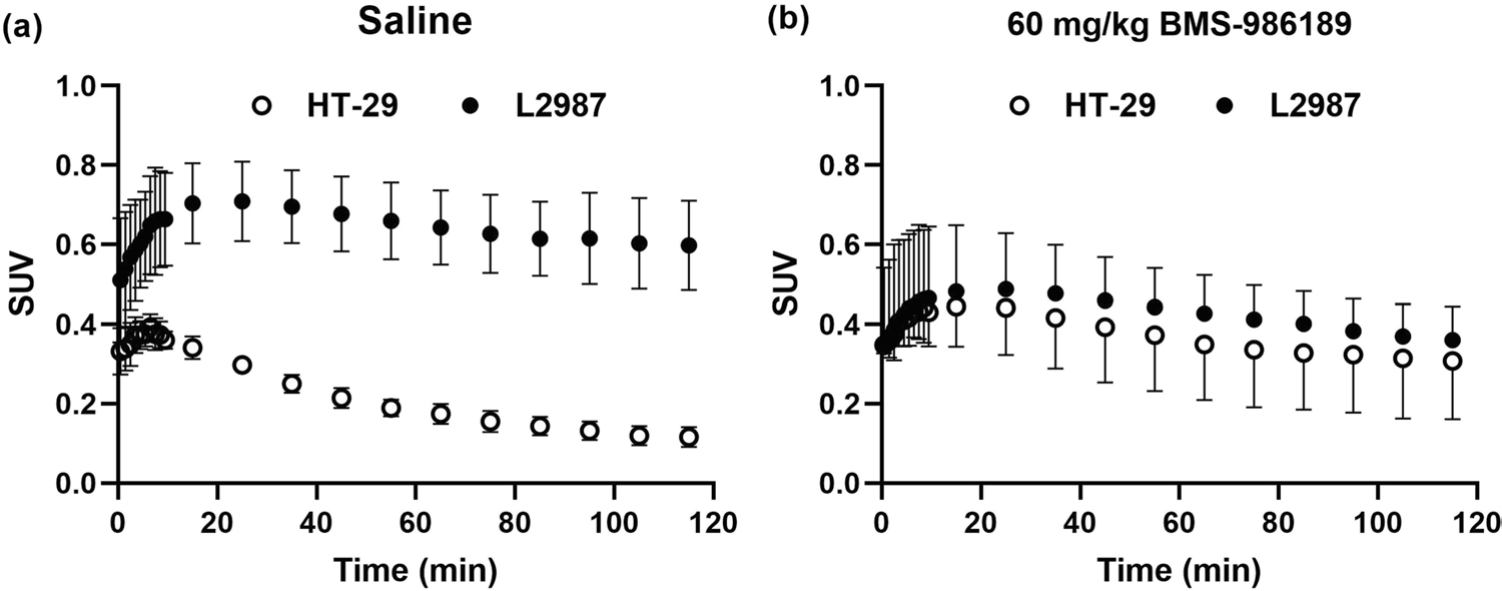
*In vivo* PET imaging was performed to assess the distribution of ^18^F-BMS-986229 in mice bearing tumor and the effect of blocking of tracer binding through BMS-986189 (60 mg/kg) administration. ^18^F-BMS-986229 uptake (SUV) into L2987 (PD-L1 +) and HT-29 (PD-L1-) tumors in mice pretreated with (**a**) saline and (**b**) 60 mg/kg BMS-986189

To evaluate the characteristics of ^18^F-BMS-986229 as a PET tracer for PD-L1, *in vivo* PET imaging was performed for the distribution of ^18^F-BMS-986229 in tumor-bearing mice and the effect of blocking of the tracer binding following BMS-986189 (60 mg/kg) administration. Each mouse received a single 60 mg/kg subcutaneous dose of BMS-986189 (*n* = 4) or saline (*n* = 4) 30 min before tracer administration via the tail vein. The resultant time-activity curves are plotted in Fig. 2, demonstrating a longer sustainable PET tracer in L2987 and rapid clearance from HT-29. *In vivo* PET imaging in mice bearing bilateral tumors demonstrated that the uptake to L2987 is higher than the value in HT-29, with a ratio of 5:1 (at 90–100 min after tracer administration, Fig. 2(A)). In Fig. 2(B), the uptake of the PET tracer in L2987 tumors was reduced by 38%, but the uptake of the PET tracer in HT-29 increased by 45% after blockade with BMS-986189.

**Fig. 3:**
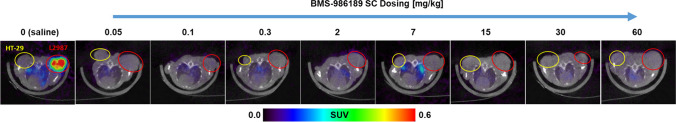
Representative PET/CT images from each dose group. Pre-dosing with BMS-986189 from 0.05 to 60 mg/kg fully displaced the uptake of ^18^F-BMS-986229 in L2987 (PD-L1 positive) tumors from the levels in the saline dosed group, with an 83.3% to 93.0% SUV decrease

**Fig. 4:**
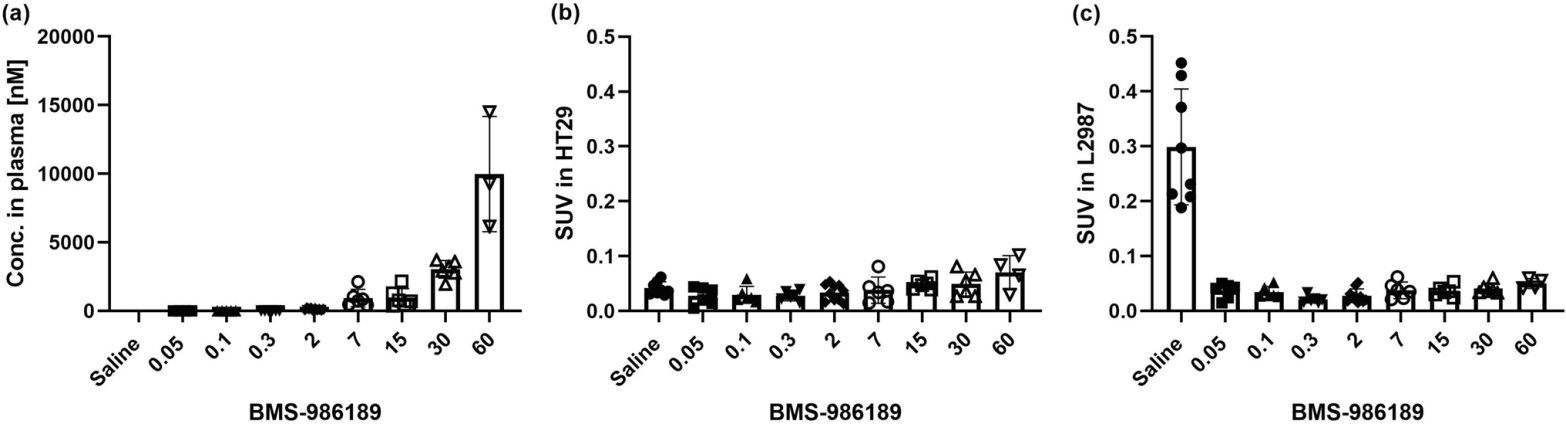
Concentrations of BMS-986189 in plasma (**a**) and SUVs in each tumor (**b** and **c**). No data are shown in a saline group of (**a**) because the values were below the limit of quantification. Three individual data are missed in the 60 mg/kg group of (**a**) due to missing blood sampling

Next, to assess the dose-dependent target engagement by BMS-986189 (0.05, 0.1, 0.3, 2, 7, 15, 30, 60 mg/kg), *in vivo* PET imaging was performed. Representative PET images for each dose group are shown in Fig. 3. The tracer SUV and plasma concentrations of BMS-986189 are shown in Fig. 4. Pre-dosing with BMS-986189 from 0.05 to 60 mg/kg fully displaced the tracer uptake in the L2987 (PD-L1 +) tumors from the levels observed in the saline dosed group, resulting in an 83%-93% reduction in SUV.

### Radiation Dosimetry in NHPs

**Fig. 5:**
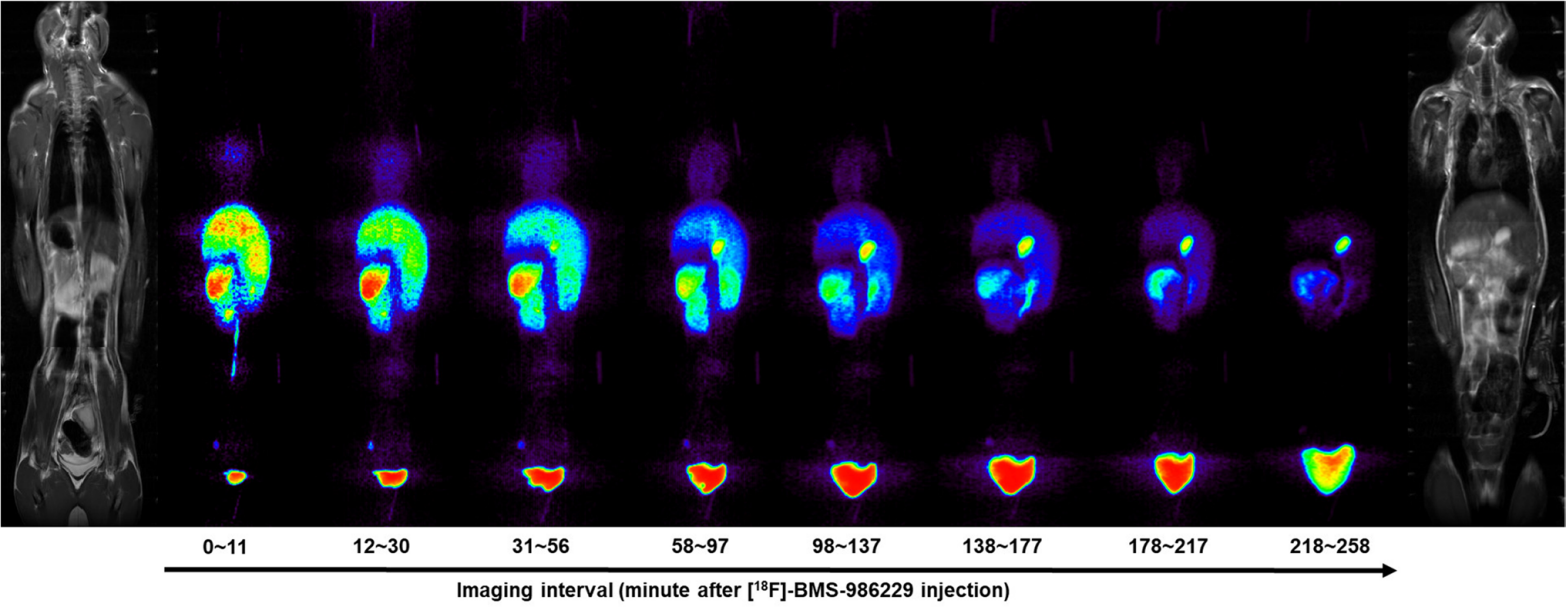
PET and MRI images in NHP. Each PET image is a non-radioactive decay corrected image

Figure 5 shows a representative coronal view of the stitched whole-body PET images in a NHP over the 4.5 h scan period. The residence times from all source organs are summarized in Table 1. The estimated absorbed radiation dose from OLINDA/EXM 1.0 was calculated and is reported in Table 2. The five organs most exposed to the PET tracer were the urinary bladder wall, the gallbladder wall, the kidneys, spleen, and liver. Under RDRC exposure limits as specified in CFR 361.1 [25], the urinary bladder wall was the dose-limiting organ, with a single study dose of 285.1 MBq (7.7 mCi) for the average subject. The estimated effective dose (0.025 mSv/MBq) for ^18^F-BMS-986229 was consistent with published values for other ^18^F tracers [26–28].

**Table 1:** Calculated organ residence time (h)

	B5153 M	B4325 M	B5056 F	B5045 F
Gallbladder	0.1057	0.0560	0.1004	0.0547
Heart	0.0298	0.0579	0.0512	0.0365
Kidney	0.1667	0.2176	0.1100	0.1150
Liver	0.3912	0.4630	0.4706	0.3609
Spleen	0.0460	0.1010	0.1006	0.0699
Bladder	0.2698	0.2814	0.4836	0.1264
Remainder body	1.6346	1.4669	1.3274	1.8803

Shown are residence time for each organ converted to human: Data for male NHPs were converted to a 74 kg human subject and female NHPs to a 57 kg human subject. *M *male, *F *female

**Table 2:** Dosimetry values in SI Units (mSv/MBq)

Target organ	B5153 M	B4325 M	B5056 F	B5045 F	Male (74 kg)	Female (57 kg)	Average subject
Adrenals	0.017	0.019	0.021	0.022	0.018	0.021	0.020
Brain	0.007	0.006	0.007	0.010	0.007	0.009	0.008
Breasts	0.008	0.008	0.009	0.011	0.008	0.010	0.009
Gallbladder wall	0.184	0.108	0.197	0.117	0.146	0.157	0.152
LLI wall	0.014	0.013	0.019	0.016	0.013	0.018	0.015
Small intestine	0.014	0.013	0.016	0.016	0.014	0.016	0.015
Stomach wall	0.013	0.014	0.016	0.017	0.013	0.017	0.015
ULI wall	0.015	0.014	0.018	0.018	0.014	0.018	0.016
Heart wall	0.018	0.024	0.026	0.024	0.021	0.025	0.023
Kidneys	0.110	0.142	0.084	0.087	0.126	0.086	0.106
Liver	0.055	0.063	0.083	0.066	0.059	0.074	0.067
Lungs	0.011	0.011	0.013	0.015	0.011	0.014	0.012
Muscle	0.010	0.010	0.012	0.013	0.010	0.013	0.011
Ovaries	N/A	N/A	0.019	0.017	N/A	0.018	0.018
Pancreas	0.018	0.020	0.023	0.023	0.019	0.023	0.021
Red marrow	0.010	0.010	0.012	0.013	0.010	0.012	0.011
Osteogenic cells	0.014	0.013	0.016	0.020	0.013	0.018	0.016
Skin	0.007	0.007	0.008	0.010	0.007	0.009	0.008
Spleen	0.055	0.111	0.132	0.095	0.083	0.114	0.098
Testes	0.010	0.010	N/A	N/A	0.010	N/A	0.010
Thymus	0.009	0.009	0.011	0.014	0.009	0.012	0.011
Thyroid	0.008	0.008	0.008	0.011	0.008	0.010	0.009
Urinary bladder wall	0.136	0.141	0.329	0.094	0.139	0.212	0.175
Uterus	N/A	N/A	0.027	0.019	N/A	0.023	0.023
Total Body	0.012	0.012	0.015	0.015	0.012	0.015	0.013
Effective dose equivalent	0.040	0.041	0.059	0.038	0.041	0.048	0.045
Effective dose	0.020	0.024	0.034	0.023	0.022	0.029	0.025

Shown are absorbed radiation dose estimates from OLINDA/EXM output. SI-Units = international system of units, *M *male, *F *female, *N*/*A *not applicable

## Discussion

According to the preclinical results reported herein, ^18^F-BMS-986229, a macrocyclic peptide PD-L1 PET tracer demonstrated a robust composite profile of binding, tumor penetration, and *in vivo* clearance. This ^18^F-macrocyclic peptide PD-L1 PET imaging enables the measurement of PD-L1 expression in tumors over the course of treatment and enables the measurement of the levels of *in vivo* sustained anti-PD-L1 checkpoint inhibitor [19]. Furthermore, ^18^F-BMS-986229 demonstrates the advantages of a shorter *in vivo* half-life and a lower molecular weight PET tracer, compared to antibody-based PD-L1 PET tracers. These advantages listed above will bring us one step closer to personalized medicine or precision medicine when we use long-axis FOV PET systems [29]. More specifically, the long-axis FOV PET imaging system appears to be able to perform multiple PET images in a short time by using a small amount of PET tracer (< 37 MBq) and shortening the scan duration (2–3 min). From the point of view of drug development, these changes will serve as an opportunity to add *in vivo* pharmacokinetics/pharmacodynamics (PK/PD) information of drugs in target tissues or lesions to the current method that relied on PK/PD in the blood of drugs. In other words, ^18^F-radiolabeled PET tracers derived directly from pharmaceuticals—checkpoint inhibitor of PD-1:PD-L1 (as a drug) and its analogs (as PET tracer imaging)—are expected to have the advantages of selecting patients, providing an opportunity to switch to alternative treatment, and predicting treatment effects. Recently, the peptide-based ^68^ Ga-WL12 was reported for preclinical imaging of PD-L1 expression in hPD-L1 and CHO tumors [30]. *Ex vivo* biodistribution analysis showed ~ ninefold increase of ^68^ Ga-WL12 accumulation in hPD-L1 + versus CHO tumors, while imaging-derived SUV analysis showed ~ sevenfold increase of ^18^F-BMS-986229 accumulation in L2987 versus HT-29 tumors. Regarding peptide-based PET tracers, both tracers provide a high affinity for PD-L1 in tumor models and improved image contrast, which will be beneficial to the routine clinical workflow.

The ^18^F-BMS-986229 PET tracer demonstrates specific binding to a variety of PD-L1-positive tissues and high affinity for PD-L1 inhibitors in development through a variety of *in vitro* and *ex vivo* autoradiographic and preclinical *in vivo* PET images. In *in vitro* autoradiography, it shows specific binding of ^18^F-BMS-986229 to PD-L1, during which ~ 90% of the autoradiography signal was blocked upon co-incubation with 1 nM BMS-986189. Another *in vitro* autoradiography of human NSCLC tumor tissue shows high-affinity binding of ^18^F-BMS-986229 to human PD-L1, which was blocked upon co-incubation with BMS-986189 in a dose-dependent manner. *In vivo* PET imaging of tumor-bearing mice shows that tracer uptake was clearly higher in PD-L1 positive tumors compared to PD-L1 negative tumors (5:1 ratio), indicating the tracer's PD-L1 binding specificity. As reported in another paper [19], ^18^F-BMS-986229 was synthesized via copper-mediated click chemistry to create a PD-L1 PET tracer with picomolar affinity suitable for evaluating PD-L1 expression. This paper also demonstrates, via PET imaging in healthy NHPs, that the same advantages of macrocyclic peptide PET tracers in producing high signal-to-noise in PD-L1 positive tissue and low background signal in non-expressing tissue, which is validated by comparing with ^89^Zr-radiolabeled PD-L1 Adnectin or mAb PET tracers.

In addition, the *in vivo* PET imaging studies conducted with multiple concentrations of BMS-986189 spanning a dose range of 0.05 ~ 60 mg/kg demonstrated up to 83%-93% reduction of SUV in PD-L1 positive tumors. These results suggest that the PET tracer is highly sensitive and prevents tracer binding even at the lowest concentration of the blocking ligand, suggesting that an 0.05 mg/kg dose of BMS-986189 (plasma conc. ≈ 0.7 nM) is sufficient to achieve plateau saturation of L2987 tumor PD-L1 receptors.

Lastly, *in vivo* PET imaging in 4 healthy NHPs was performed to estimate the human absorbed dose in normal tissues. These preclinical dosimetry estimates indicated that ^18^F-BMS-986229 can be safely used as a PET radiotracer in humans. The results suggest that the effective dose and effective dose equivalent are comparable to other ^18^F tracers that are routinely utilized in clinical study. Within the suggested dose limits, the absorbed radiation dose for all organs would be expected to be below established RDRC guidelines (21CFR361.1) and generally recognized as safe for both a single IV administration as well as a cumulative yearly dose. To confirm the safety profile, dosimetry can be calculated in the initial human subjects to refine the estimates provided here.

Note that, human clinical study results for the ^18^F-BMT-986229 PET tracer within the prospective ADAPT-IT unresectable melanoma trial were recently disclosed and demonstrated a good safety and tolerability, while PD-L1 positivity by PET imaging at baseline appeared to be associated with early efficacy from nivolumab + ipilimumab in this small patient cohort [31]. In addition, the safety and feasibility of whole-body PD-L1 assessment using ^18^F-BMT-986229 PET were also confirmed in patients with esophageal, stomach, or gastroesophageal junction cancer, and the authors reported a strong correlation between the most avid lesion on PET with PD-L1 combined positive score [32].

## Conclusion

This study demonstrates the strong potential of ^18^F-BMS-986229 as a macrocyclic peptide PD-L1 PET tracer for the assessment of tumor PD-L1 expression. The compound exhibits high *in vivo* uptake to PD-L1 positive tumors, low background signals, and a short blood half-life supportive of same day imaging in the clinic. In addition, the results of radiation dosimetry indicate that the PET tracer is safe for clinical use. Clinical studies with ^18^F-BMS-986229 are currently underway to further evaluate the performance of the tracer in oncology patients undergoing treatment with immune checkpoint inhibitors.

## References

[1] Keir ME, Butte MJ, Freeman GJ (2008). PD-1 and its ligands in tolerance and immunity. Annu Rev Immunol.

[2] Pardoll DM (2012). The blockade of immune checkpoints in cancer immunotherapy. Nat Rev Cancer.

[3] Bordon Y (2014). Checkpoint parley. Nat Rev Cancer.

[4] Leung D, Bonacorsi S, Smith RA (2021). Molecular imaging and the PD-L1 pathway: from bench to Clinic. Front Oncol.

[5] Festino L, Botti G, Lorigan P (2016). Cancer treatment with anti-PD-1/PD-L1 agents: Is PD-L1 expression a biomarker for patient selection. Drugs.

[6] Brahmer JR, Hammers H, Lipson EJ (2015). Nivolumab: targeting PD-1 to bolster antitumor immunity. Future Oncol.

[7] Forde PM, Lu SS, Provencio M (2022). Neoadjuvant Nivolumab plus chemotherapy in resectable lung cancer. N Engl J Med.

[8] Twomey JD, Zhang B (2021). Cancer Immunotherapy update: FDA-approved checkpoint inhibitors and companion diagnostics. AAPS J.

[9] Herbst RS, Soria J-C, Kowanetz M (2014). Predictive correlates of response to the anti-PD-L1 antibody MPDL3280A in cancer patients. Nature.

[10] Taube JM, Klein A, Brahmer JR (2014). Association of PD-1, PD-1 ligands, and other features of the tumor immune microenvironment with response to anti-PD-1 therapy. Clin Cancer Res.

[11] Topalian SL, Hodi FS, Brahmer JR (2012). Safety, activity, and immune correlates of anti-PD-1 antibody in cancer. N Engl J Med.

[12] Chatterjee S, Lesniak WG, Miller MS (2017). Rapid PD-L1 detection in tumors with PET using a highly specific peptide. Biochem Biophys Res Commun.

[13] England CG, Ehlerding EB, Hernandez R (2017). Preclinical pharmacokinetics and biodistribution studies of ^89^Zr-labeled pembrolizumab. J Nucl Med.

[14] Lesniak WG, Ghatterjee S, Gabrielson M (2016). PD-L1 detection in tumors using ^64^Cu-Atezolizumab with PET. Bioconjug Chem.

[15] Maute RL, Gordon SR, Mayer AT (2015). Engineering high-affinity PD-1 variants for optimized immunotherapy and immuno-PET imaging. Proc Natl Acad Sci USA.

[16] Mayer AT, Natarajan A, Gordon SR (2017). Practical immuno-PET radiotracer design considerations for human immune checkpoint imaging. J Nucl Med.

[17] Donnelly D, Smith RA, Morin P (2018). Synthesis and biologic evaluation of a novel ^18^F-labeled adnectin as a PET radioligand for imaging PD-L1 expression. J Nucl Med.

[18] Huisman MC, Niemeijer AN, Windhorst AD (2020). Quantification of PD-L1 expression with ^18^F-BMS-986192 PET/CT in patients with advanced-stage non-small cell lung cancer. J Nucl Med.

[19] Donnelly DJ, Kim J, Tran T (2023). The discovery and evaluation of 18F-BMS-986229, a novel macrocyclic peptide PET radioligand for the measurement of PD-L1 expression and in-vivo PD-L1 target engagement. Eur J Nucl Med Mol Imaging.

[20] Loening AM, Gambhir SS (2003). AMIDE: a free software tool for multimodality medical images analysis. Mol Imaging.

[21] Cristy M, Eckemrman KF (1987) Specific absorbed fractions of energy at various ages from internal photon sources. I. Methods. Oak Ridge National Laboratory ORNL/TM-8381/V1

[22] Stabin MG, Sparks RB, Crowe E (2005). OLNIDA/EXM: The second-generation personal computer software for internal dose assessment in nuclear medicine. J Nucl Med.

[23] Seneca N, Skinbjerg M, Zoghbi SS (2008). Kinetic brain analysis and whole-body imaging in monkey of ^11^C-MNPA: a dopamine agonist radioligand. Synapse.

[24] Sprague DR, Fujita M, Ryu YH (2008). Whole body biodistribution and radiation dosimetry in monkeys and humans of the phosphodiesterase 4 radioligand [^11^C](R)-rolipram: comparison of two-dimensional planar, bisected and quadrisected image analyses. Nucl Med Biol.

[25] Radioactive drugs for certain research use (2016) Title 21, Chapter I, Subchapter D, Part 361. C.F.R, 361.1

[26] Kimura Y, Simeon FG, Hatazawa J (2010). Biodistribution and radiation dosimetry of a positron emission tomographic ligand, ^18^F-SP203, to image metabotropic glutamate subtype 5 receptors in humans. Eur J Nucl Med Mol Imaging.

[27] Andersson M, Johansson L, Mattsson S (2016). Organ doses and effective doses for five PET radiopharmaceuticals. Radiat Prot Dosimetry.

[28] Nag S, Fazio P, Lehmann L (2016). In vivo and in vitro characterization of a novel MAO-B inhibitor radioligand, ^18^F-labeled deuterated fluorodepernyl. J Nucl Med.

[29] Pantel AR, Mankoff DA, Karp JS (2022). Total-body PET: Will it change science and practice?. J Nucl Med.

[30] De Silva RA, Kumar D, Lisok A (2018). Peptide-based ^68^Ga-PET radiotracer for imaging PD-L1 expression in cancer. Mol Pharm.

[31] Postow MA, Mauguen A, Frosina D et al (2022) Assessing PD-L1 without a biopsy and through PD-L1 PET imaging with ^18^F-BMS-986229. J Clin Oncol 40(16_suppl). Abstract #2578

[32] Cytryn S, Lumish M, Paroder V et al (2022) Feasibility, safety, and biodistribution of ^18^F-BMS-986229 PET in patients with esophagogastric (EG) cancer. Ann Oncol 33:S335, P-244

